# Caught in a no-win situation: discussions about CCSVI between persons with multiple sclerosis and their neurologists – a qualitative study

**DOI:** 10.1186/s12883-017-0954-7

**Published:** 2017-09-07

**Authors:** S. Michelle Driedger, Ryan Maier, Ruth Ann Marrie, Melissa Brouwers

**Affiliations:** 1 Department of Community Health Sciences, Max Rady College of Medicine, Rady Faculty of Health Sciences, University of Manitoba, Winnipeg, MB Canada; 2 Departments of Internal Medicine and Community Health Sciences, Max Rady College of Medicine, Rady Faculty of Health Sciences, University of Manitoba, Winnipeg, MB Canada; 3 Department of Oncology, McMaster University, Hamilton, ON Canada

**Keywords:** Chronic disease, Communication, Chronic Cerebrospinal Venous Insufficiency, Liberation therapy, Canada, Clinical interactions, Venous angioplasty

## Abstract

**Background:**

In recent years, shared decision making (SDM) has been promoted as a model to guide interactions between persons with MS and their neurologists to reach mutually satisfying decisions about disease management – generally about deciding treatment courses of prevailing disease modifying therapies. In 2009, Dr. Paolo Zamboni introduced the world to his hypothesis of Chronic Cerebrospinal Venous Insufficiency (CCSVI) as a cause of MS and proposed venous angioplasty (‘liberation therapy’) as a potential therapy. This study explores the discussions that took place between persons with MS (PwMS) and their neurologists about CCSVI against the backdrop of the recent calls for the use of SDM to guide clinical conversations.

**Methods:**

In 2012, study researchers conducted focus groups with PwMS (*n* = 69) in Winnipeg, Canada. Interviews with key informants were also carried out with 15 participants across Canada who were stakeholders in the MS community: advocacy organizations, MS clinicians (i.e. neurologists, nurses), clinical researchers, and government health policy makers.

**Results:**

PwMS reported a variety of experiences when attempting to discuss CCSVI with their neurologist. Some found that there was little effort to engage in desired discussions or were dissatisfied with critical or cautious stances of their neurologist. This led to communication breakdowns, broken relationships, and decisions to autonomously access alternative opinions or liberation therapy. Other participants were appreciative when clinicians engaged them in discussions and were more receptive to more critical appraisals of the evidence. Key informants reported that they too had heard of neurologists who refused to discuss CCSVI with patients and that neurology as a whole had been particularly vilified for their response to the hypothesis. Clinicians indicated that they had shared information as best they could but recommended against seeking liberation therapy. They noted that being respectful of patient emotions, values, and hope were also key to maintaining good relationships.

**Conclusions:**

While CCSVI proved a challenging context to carry out patient-physician discussions and brought numerous tensions to the surface, following the approach of SDM can minimize the potential for unfortunate outcomes as much as possible because it is based on principles of respect and more two-way communication.

**Electronic supplementary material:**

The online version of this article (10.1186/s12883-017-0954-7) contains supplementary material, which is available to authorized users.

## Background

### The nature of multiple sclerosis

Canada has among the highest prevalence of multiple sclerosis (MS) in the world, with an estimated prevalence of over 250 per 100,000 of the population [1]. This translates into approximately 100,000 Canadians living with the disease [2]. MS is an incurable chronic immune-mediated disease which affects the brain, optic nerves, and spinal cord [3]. Symptoms vary from person to person [4], and while the clinical course can be broadly classified into several groups [5], the progression of MS is unique for each individual, and typically worsens over time. In terms of disease management, there is an increasing array of disease modifying therapies (DMTs) that partially reduce the risk of relapses and slow disease progression, but they have varying benefit-risk profiles [6, 7]. The inherent uncertainty of MS outcomes and its treatments, plus its chronic and degenerative nature, make living with MS an emotional experience for persons with MS (PwMS) and their families [8].

### MS care in Canada

Canada has a universal, publicly funded health system which provides access to medically necessary hospital and physician services [9]. Health services delivery is the responsibility of Canada’s provinces and territories. Specialized MS Clinics exist across the country. These clinics offer expertise in the diagnosis and treatment of MS. Care is delivered by teams involving neurologists, nurses, and allied health professionals such as occupational therapists and physical therapists; the specific resources available and roles of specific professionals may differ somewhat from one clinic to another. In Manitoba, the Winnipeg MS Clinic is the sole clinic providing specialized MS care, and provides access to neurologists, nurses, a physiotherapist, occupational therapist, social worker and dietitian. In most provinces, prescription of disease-modifying therapies is limited to neurologists with specific expertise in MS. Provincial programs provide coverage for these disease-modifying therapies; however, criteria for accessing these therapies and residual out of pocket costs vary from one province to another [10].

### Recommendations for shared decision making (SDM) in the MS context

The relationship that PwMS have with their MS specialist, typically their neurologist, is a core foundation for MS disease management. Indeed, many PwMS have reported having good confidence in their neurologist’s care and that their concerns are being heard and addressed [11]. However, these often long-term relationships have also been shown to be sources of considerable conflict and dissatisfaction for many other PwMS, where sub-optimal perceptions of communication and information exchange have been most commonly identified problem areas [12–14]. PwMS have expressed their desire for more active or autonomous roles in making decisions about disease management and therapies arising from perceptions that some clinicians had often taken a paternalistic approach and gave limited or selective information while still expecting PwMS to adhere to physician recommendations [14–18]. Furthermore, some MS doctors overlook the emotional cues (e.g. worry, anxiety) of their MS patients [8].

In 2007, Heesen et al. [19] declared that MS was a “prototypic condition” (and later, a “paradigmatic disease” [20]) for using a shared decision making (SDM) process between PwMS and their neurologists to reach mutually satisfying decisions about disease management – generally about deciding on which DMTs to use. To address the sources of conflict noted above, SDM promotes a more egalitarian relationship, an openness of dialogue, and a dedication to fulsome information exchange. Both parties bring to the discussion their own set of required competencies. Clinicians are to present a set of options for DMTs, detailing their respective profiles of benefits and risks that have been established through randomized control trials (RCTs). PwMS are to share their values and risk sensibilities [19]. Both then discuss these factors while weighing the options to assist PwMS in deciding ultimately what is best for them at the time. By fostering this dialogical approach, a key aspect of SDM is that it intends to create the space for greater patient input and autonomy in decision making [21], while also impressing on doctors the need to be more empathetic to the values and personal inclinations of their patient [8]. SDM may also help to foster trust between a patient and their doctor [22–24], the loss of which could lead to a troubling breakdown of communication. The consequences of a communication breakdown could be especially problematic if PwMS then feel compelled to make decisions about disease management without consulting their neurologist, such as stopping or altering their treatment courses of DMTs or accessing alternative therapies. Altogether, the ideal outcomes of SDM are not dependent on the specific option chosen, but that decisions satisfy all parties and build trust and mutual understanding.

### Chronic Cerebrospinal Venous Insufficiency (CCSVI) and venous angioplasty

In 2009, Dr. Paolo Zamboni introduced the world to his hypothesis of CCSVI as a cause of MS and proposed venous angioplasty as a potential therapy [25, 26]. Although Zamboni’s research was based on a non-randomized small sample of PwMS, and subsequent evidence was purely anecdotal at the time, the possibility that a cause and even a cure had been found for MS gave considerable hope to many PwMS and their families. The surprising news attracted the immediate attention of Canadian media, who played a key role in raising the profile of Zamboni’s hypothesis and in highlighting the anecdotal success stories – often at the expense of more critical or scientifically rigorous perspectives (and is the subject of forthcoming papers related to this study) [27–29].

The earliest news media dubbed MS-related venous angioplasty the “liberation treatment” or “liberation therapy” [29]. While the term “liberation” can refer in a technical sense to the medical procedure of venous angioplasty proposed to restore blood flow through stenosed jugular veins, it may also (intentionally or not) bring with it emotionally charged, hope-driven desires for freedom – in this case, for a person to be “liberated” from their MS to some degree. Thereafter, in traditional media, but also especially in new forms of social media (blogs, networking sites), issues often became framed in normative terms of rights (for PwMS who were being ‘denied’ a breakthrough therapy) and in journalistic narrative tropes as ‘good guys’ and ‘underdogs’ (PwMS and advocates for CCSVI and liberation therapy) versus the ‘bad guys’ and ‘villains’ (scientists and MS clinicians who were cautious about the hypothesis) [27, 30–34]. Moreover, the quality and reliability of evidence for CCSVI and liberation therapy found on the internet and social media is not always self-apparent or easy to contextualize, which often helped to blur the lines between anecdotal and scientifically validated kinds of evidence [33].

PwMS have been recognized as highly interested, sophisticated, and resourceful information seekers and informal researchers about their disease as they follow the latest research to assess what might be useful for their disease management [35–39]. Although Zamboni’s treatment still required extensive clinical trials to investigate its efficacy and safety (and a link between CCSVI and MS and in the efficacy of venous angioplasty still remains to be proven even after a number of follow-up studies [40–42]), many Canadian PwMS became immediately interested in the potential breakthrough and began demanding domestic access to this as a publicly funded treatment or at least access to diagnostic testing for ‘collapsed veins.’ However, without the necessary evidence base to demonstrate substantial positive benefit, the publically funded Canadian health system could not justify supporting a hypothetical treatment as an insured service [27]. Facing a seemingly recalcitrant or indifferent health system, and despite a lack of evidence, many PwMS felt compelled by their hope to travel abroad to have their veins tested and receive venous angioplasty, often paying large sums of money as ‘medical tourists’ [43].

This paper explores the discussions that took place between PwMS and their neurologists following the release of news of Zamboni’s hypothesis. We investigate these conversations about CCSVI against the backdrop of the recent calls for SDM to improve communicating and decision making about therapeutic options. However, it is crucial to recognize that the contexts of CCSVI and Canada’s healthcare system represent potential confounders for considering this a ‘prototypical’ or ‘paradigmatic’ situation for following a SDM approach in the MS clinical setting. Notably, SDM was proposed for scenarios of DMTs that had much more clearly defined risk-benefit profiles and evidence bases derived from prevailing scientific standards of RCTs. Furthermore, testing and treatment for CCSVI was not available to PwMS in Canada, and therefore it was not even a potential domestic option for clinical recommendations. Nevertheless, it is precisely with these confounders in mind that we will examine the appropriateness, applicability, and suitability of following the general principles of SDM in this context – because regardless of the state of the evidence or the unavailability of the treatment in Canada – PwMS still went to their neurologists to discuss CCSVI and many still wanted access to its testing and treatment. We are interested in the consequences for patient-physician dynamics, relationships, and outcomes, if neurologists reported they attempted to apply principles of SDM during this controversial time – and, conversely, the repercussions if they did not. Investigating a scenario where the issue in question and the SDM approach do not fit together cleanly (or as ideally intended) is important and still timely, because a debate over a potentially ‘breakthrough’ therapy, procedure, drug, etc. – whether for MS or some other disease – is certainly a situation likely to happen again in the future. Under such circumstances, clinicians and patients (and other stakeholders) will have to grapple with how to make decisions when the evidence is lacking, uncertain, or contested, and yet people may still demand access (at the most) or more information (at the least) from their health care provider and the health system. This study thus holds some potential to provide some insights into how to better navigate such scenarios.

While there have been some explorations into the broader public discourse among the MS community and other media stakeholders when news of CCSVI went public, there has yet to be a critical examination of conversations between PwMS and their physicians that the controversial treatment engendered. This study seeks to fill this gap by investigating the perspectives a variety of MS stakeholders, including PwMS, MS clinicians, advocacy organizations, researchers, and government health policy makers. While this study is focused largely on the interactions between PwMS and their neurologist, relevant non-neurologist key informant perspectives are also included as they were also part of, or privy to, the broader clinical and popular discourses within which these relationships were embedded.

## Methods

For this study, in 2012 researchers conducted seven focus groups with 69 PwMS in Winnipeg, Manitoba, who were recruited from the local MS clinic. Focus groups were made up of people with different types of MS (relapsing-remitting, and primary or secondary progressive) who had lived with their diagnoses for different lengths of time ranging from less than 5 years, 10 to 19 years, to over 20 years (due to scheduling and availability, sometimes a participant had to join a focus group that was outside of their own disease duration range). Focus groups lasted approximately 2 h. The interview guide was pilot tested with the first focus group (with participants of varying disease type and duration). As no changes were required, findings from the pilot test were included in the analysis. Some participants were accompanied by their spouse who also contributed to the discussions to aid with communication as well as to reflect on the impact of MS for the entire family. All participants gave informed consent and PwMS were given an honorarium of $60 for participating in the research project. Discussions were semi-structured and participants were asked to give their thoughts on a variety of MS-related issues, including their information-seeking behaviors and information sources consulted, the CCSVI hypothesis and venous angioplasty, and their communication interactions with their doctors about MS.

Between 2012 and 2014, researchers also conducted key informant interviews with 15 participants who were stakeholders in the MS community when the CCSVI theory emerged. Seven neurologists were interviewed and the rest were members of advocacy organizations, other types of clinicians who worked primarily in the MS field (i.e. nurse clinicians, physiatrists, psychiatrists), clinical researchers, and government health policy makers. Interviews lasted one to two hours and were semi-structured. Participants were asked about their views on CCSVI and venous angioplasty, and clinicians were asked about the discussions they had with their patients about CCSVI. Non-clinicians were also asked about their second-hand impressions of doctor-patient interactions of that time. In neither the focus group interviews nor the key informant interviews were participants asked explicitly about shared decision making. Rather, participants self-reported on the nature of the discussions they had with health care providers and/or PwMS regarding MS care and the issue of CCSVI.

Following data collection, recorded interviews were transcribed, audio-verified and imported into NVivo10, a qualitative data analysis software. Coding schemes were developed by SMD and three research assistants by using an iterative process of reviewing transcripts and identifying key themes (e.g. doctor-patient communication) in the data using constant comparative and concept-development approaches [44]. Data were coded and a sample inter-coder reliability test achieved 86% Kappa scores, well above recommended levels [45]. In the results that follow, key issues around doctor-patient communication in the context of CCSVI and venous angioplasty will be presented and representative quotes will be used to illustrate common themes that were raised during the conversations. To protect anonymity, PwMS (and their spouses) are given a pseudonym and key informants are referred to only by their status as a neurologist or a non-neurologist MS clinician, or by their associated organization.

An additional file provides a more detailed methods description, with a particular emphasis on the analysis process (see Additional file 1) as well as copies of the focus group (see Additional file 2) and key informant (see Additional file 3) interview guides. Also included with this article is a checklist of consolidated criteria for reporting qualitative research (COREQ). This checklist is used when reporting qualitative research to ensure greater transparency around data collection, analysis, and reporting processes (see Additional file 4).

## Results

### PwMS: Description of focus group participants

Twelve participants had elected to travel out of Canada and already received the liberation therapy at the time the focus groups were conducted (June 2012). All but one who had sought out the treatment had lived with the disease for more than 10 years, and 8 had the most debilitating form of MS: primary or secondary progressive. There existed a spectrum among all participants in their opinions about CCSVI and liberation therapy. Some were very skeptical about it, some wanted to wait to see what research would show, and others were much more supportive of it – including those who had gotten the treatment. Despite some skepticism that existed among participants in all focus groups, there tended to be a common motivation to positively view the potential of the CCSVI hypothesis – at the time it gave many PwMS and their families a source of much needed hope for a better future. This positive perception of CCSVI was more general to PwMS who had MS for a long period of time and who had more debilitating progressive forms of the disease – although some still expressed skepticism or even pessimism towards it. On the other hand, while patients with a recent diagnosis and with little progression of their MS were more likely to view the new hypothesis cautiously, there were also some who were more enthusiastic about the treatment’s potential.

Most of the participants had done at least some of their own independent research into CCSVI and venous angioplasty. Some participants reported that they sought out information from traditional mainstream media sources (television, newspaper), and others went to greater lengths to find information, whether doing Google searches, or looking to websites of MS stakeholders (eg. MS Society, Dr. Paolo Zamboni’s website, advocate or community-driven websites or blogs, social media sites like Facebook, etc.), or consulting academic journals. Nevertheless, with as much information they could locate from their own research, many participants went to their MS clinician and asked them about the hypothesis and the treatment.

### Focus group participants’ perspectives of their discussions with their doctors about CCSVI and venous angioplasty – communication breakdowns

In all focus groups, many PwMS recalled their experiences with their neurologists with a tone of discouragement when they had attempted to discuss CCSVI and venous angioplasty. While we found that some participants shared positive remarks about their conversations with neurologists (see below), those voices were often overwhelmed by other negative comments. Some PwMS indicated that their neurologist was not even willing to indulge in a conversation about CCSVI and venous angioplasty. For other participants, they felt that the information that they got from their neurologist about liberation therapy was decidedly negative. They felt that their desire to discuss CCSVI was met with immediate skepticism, derision of the hypothesis, or prompt recommendations against seeking venous angioplasty without any further discussion. For many of these participants, such responses from their MS clinician all fed into a generalized view that neurologists seemed disinterested in, totally opposed to, or implicitly biased against a new therapy that already looked like it was producing positive results. The field of neurology as a whole was similarly characterized in negative tones.

But [my neurologist] doesn’t even want to talk about it [CCSVI]. It’s just like it’s taboo. [Barbara, 20+ years]

They [healthcare providers] are all comfortable [talking about CCSVI and venous angioplasty] except the neurologists. [Leo, husband of Elaine, 20+ years]

They [the neurologists] were the naysayers [about Zamboni’s hypothesis]. [Gord, husband of Lisa, 20+ years]

All [neurologists were] negative. [Clint, husband of Martha, 10-19 years]

Refused a discussion of the new hypothesis, PwMS became frustrated because they desperately wanted any or as much information they could get. This frustration often became directed at their neurologists, who were seen as gatekeepers to more information about CCSVI as well as to potential access to testing and treatment. Many simply wanted some support or respectful dialogue in whether they should be making a decision about whether to travel to get venous angioplasty.

#### CCSVI as a source of hope for PwMS

At times, PwMS linked their dissatisfaction about their discussions with their neurologists about CCSVI with broader concerns they had about communication with, and the care by, their neurologists.

My neurologist told me they didn’t agree with the CCSVI treatment. But then in the next breath told me that I’d be blind in a wheelchair. And I think, I personally feel a lot—I’m not saying all—a lot of neurologists are very negative when they speak to MS patients and they give you no hope, no hope. I’ve seen three neurologists since 19 and a half years ago and the first one told me the same thing that I’d be blind in a wheelchair. And I never went back to them. I find a lot of neurologists aren’t supportive. [Pat, 10-19 years]

The sense of hope that the prospective hypothesis offered was often more pronounced for those PwMS who had lived with MS for many years or who saw themselves as further along on an irreversible downward slope of MS progression. For them, venous angioplasty seemed to offer the last chance to potentially halt or reverse some of the symptoms of their disease and to reclaim lost or cherished abilities. Instead, they felt that not even the hope of a potential new understanding of their disease and its treatment was being afforded to them or even acknowledged by their care providers. For these participants, it did not bother them that there was some risk or possible lack of effectiveness to the unproven therapy. In fact, for some the uncertainty surrounding venous angioplasty was in the same vein as that which also characterizes the uncertain efficacy of approved and available DMTs.

I’m progressive and every year I’m going down that ramp. And pretty soon I’m not going to be able to do the things … I can’t get down on my hands and knees and play with my grandson. [Hank, 10-19 years]

I really want to have [venous angioplasty] done regardless of the outcome. I’ll try anything to be able to walk. My big thing in life is to get into my son’s house, get up the stairs, and see my grandchildren’s bedrooms. I can’t do any of that. That’s my aim. So I definitely want to have [liberation therapy] done. [Wanda, 7 years]

I had one friend of mine that went and [venous angioplasty] did absolutely nothing. I had another friend and they’re improving all the time. So it’s one of those things you take. Betaseron doesn’t help you or it does help. [Liberation therapy] might help you, it might not. The last time I saw my doctor I said my right hand is getting worse, now I don’t have a lot of function left. And they said, ‘Well, can’t do nothing for you.’ I said, ‘do you want me to just sit here and watch it happen?’ And they said, ‘yeah, basically.’ So I decided I’m going for liberation [therapy]. I wouldn’t mind getting back a little balance and stability. I wouldn’t mind anything because there’s so many symptoms that I have that even if two out of 100 get better I’m willing to go. [Hannah, less than 5 years]

This sense of hope, and lack of results from existing therapies, led many participants to wish that their neurologist would be more open to discussing or exploring new or unorthodox hypotheses like CCSVI. Sometimes they referred to particular neurologists who they felt were more ‘open-minded’ or who seemed more empathetic to the plights of their patients. Many were also adamant that they did not see venous angioplasty as a cure for their disease, but as a promising means to achieve some relief or respite from at least some of their MS symptoms or progression. Many acknowledged that there was still much more to be learned about CCSVI and that there were risks, but the positive anecdotal reports of symptom relief – even if however temporary – should be enough to prove to neurologists and the scientific community that some kind of connection existed between CCSVI and MS symptoms and that this warranted a more open-minded approach.

Our best neurologist was [Dr. X]. [They] looked like they wanted to open the door to try something. [They] had compassion, and I’ve never met a better neurologist. All the other ones are so closed-minded. [Lynn, 10-19 years]

I think [CCSVI and venous angioplasty] is phenomenal, it is going in the right direction. No, it isn’t the cure; there is still a lot of grey area. [Nina, 10-19 years]

While many participants felt that their hope was being unjustly disregarded, others who were actually comfortable with their neurologist’s skepticism of the new hypothesis agreed that a core problem was that neurologists just generally lack a capacity for good communication and compassionate care. This is also illustrative of the complexity and dynamics inherent in these relationships that debates like CCSVI can bring to the fore. People may still (have to) trust their clinician, but an underlying issue is reaffirmed.

They [neurologists] are not “people” people. I mean my neurologist is an excellent [physician] and I wouldn’t want anyone else handling my brain. [Their] bedside manner is – I mean this [referencing a soft drink can] has got a better bedside manner than my neurologist so someone has to do more. The skills they have are not [bedside manner]. [Perry, unknown years]

#### Fallout from communication breakdowns

Without their MS clinician’s desired support in providing information (at the least) or endorsing the therapy (at the most), many PwMS saw these as ultimate signs that their MS clinician did not care about their concerns. Some participants simply began avoiding talking about the new research with their neurologist and sought information elsewhere. Other participants noted that they found a more receptive medical opinion when they talked to their general practitioner rather than a MS clinician. Some also wished that their own neurologists would share the same openness and willingness to talk about a new therapy that other doctors had – even other neurologists about which they heard – seemed to offer.

I’ve never talked to [the neurologist at the MS clinic] about it [CCSVI and venous angioplasty] because I already knew where [they] stood … I had done my own research. [Marge, unknown years]

I know what you guys are saying about the neurologist. I took all the information that I had compiled through friends and family, that entire shoebox. And I took it in and tried to go through it with my neurologist at the MS clinic. And as soon as [they] saw what was contained in that shoebox [they] almost pushed it off the desk back into my lap. They said, “I don’t want to deal with this.” It upset me a lot. Whereas when I took it to my GP he went through it with me. [June, 20+ years]

My [general practitioner] said “you get the information and come to me and we’ll go through it and I’ll give you the medical perspective and then we can make the decision.” And it’s, that’s the kind of doctor we need to have. I was very, very fortunate. [Sue, 20+ years]

Some PwMS then felt compelled to then take matters into their own hands and make decisions about their MS care completely on their own. Some of those who opted to seek venous angioplasty outside of Canada – as well as many of those considering it – indicated that they did not discuss any of their independent research about it or their related decisions with their MS clinician. In some cases, if PwMS disagreed with the stance that their neurologist had, those participants simply did not tell them about their decision to attempt a major act of autonomous disease management.

But we said to the neurologist that we were seeing at the time, “What would you do?” And [they] said “I’d wait” [to get venous angioplasty] and I said “We don’t have five or seven years to wait anymore and if there is a very slim or slight [chance that it could help].” I never did tell them that we were already in the process of scheduling the trip. I just wanted their opinion what they thought about it. [Leo, husband of Elaine, 20+ years]

Some participants chose to keep their decisions to travel for venous angioplasty a secret lest it garner disapproval from their neurologist who had recommended against it. They worried that sharing their decision to get the treatment would negatively influence their relationships with their neurologist before and after they got back and that their doctors would make them feel guilty or treat them worse. Participants also expressed worry that their neurologist would no longer want to continue care for them upon their return.

In some cases, the perceived mismatch between the wishes of PwMS and the lack of desired engagement by MS clinicians led to other drastic actions with long term and significant implications. If PwMS no longer trusted their MS clinician to act in their best interest, it affected other (non-CCSVI) aspects of care, such as decisions to remain on current disease management strategies and medications. Or, going to greater lengths, some participants indicated that they may no longer want the care of a neurologist.

My doctor refused to do any testing on the veins for me and with the MS Clinic I’ve been on every kind of needle they’ve had. The pain got so severe I was on morphine. I was a drug addict for a couple of years and missed a couple of years of my life. And then when they refused to do testing on my veins I just stopped everything, I stopped everything. I said no more needles for MS, no more morphine, no more anything. No more, I want to live my life. [Clair, 20+ years]

I don’t know that I’m going to continue going to a neurologist. [Leigh, 20+ years]

#### Rationale for clinicians’ opinions

Many participants shared reasons why they thought that their neurologists were dismissive of CCSVI and venous angioplasty or why they were so unwilling to discuss or endorse it. Some attributed it to pre-existing communication problems that they had with their doctors, to a lack of empathy, or to paternalistic or patronizing indifference. Many others felt that their neurologists were against any new kinds of treatment or were inappropriately dismissive of a potentially promising new way of viewing MS and its treatment. Some PwMS believed that certain younger neurologists seemed more receptive or open to discussing the hypothesis, while older ones who had practiced a long time had perhaps become too set in their ways and were not interested in anything that challenged the status quo. More commonly, some participants noted that because CCSVI is more within the realm of vascular science, neurologists would naturally dismiss or challenge the hypothesis because MS had been traditionally thought of as a purely neurological disease. This notion fed suspicions that the CCSVI debate exposed a kind of ‘turf war’ between specialists, with neurologists trying to shut down any potential opposition to their traditional professional primacy in MS care. Some PwMS also expressed worry that neurologists may have conflicts of interests in being too involved with, or receiving financial incentives from, the pharmaceutical companies that make DMTs.

I mean, how is a neurologist going to be involved with this, it’s not their field. You need the cardiovascular surgeons and specialists involved with this because that’s their field. [June, 20+ years]

But I read that Maclean’s I think it’s May or June 2010 where they really make a case that neurologists are in league with Big Pharma. [Heather, 10-19 years]]

And [my neurologist] was dead set against it [venous angioplasty]. So, like that said a lot of things like that [my neurologist] didn’t want something that wasn’t pharmaceutically financed. [Seth, 20+ years]

#### Positive impressions of their discussions with MS clinicians

Not all of the participants’ neurologists were evasive about discussing the prospective treatment and not all PwMS were displeased with a more skeptical view of CCSVI that their neurologists shared. On the contrary, some participants pointed out that while their neurologists did not endorse or recommend venous angioplasty, they provided a more balanced scientific perspective about the existing evidence, or had at least pointed to where one could find it, and had affirmed their patient’s right to make their own decision. Many times such efforts to engage with PwMS’ concerns were generally welcomed and well received. Further, while some participants agreed with their clinician to wait for further evidence, others who still wanted venous angioplasty appreciated that their doctors were understanding enough to provide information and support them in their decision no matter which way they chose. Some participants noted that their neurologists’ skepticism was more indicative of due diligence in being cautious due to the lack of evidence – all that their doctors could really do was to recommend a ‘wait and see’ with respect to future research.

I asked my neurologist and was told it’s my choice if I want to go with it. [My neurologist] said, “Information is out there. It’s your decision. I’m not going to tell you which way to go.” [Allen, less than 5 years]

[T]here was one other MS doctor I talked to who said ‘we don’t see it [the evidence] and we’re not here to do any harm; we don’t want to hurt anyone, we’re not trying to stop people.’ And I think this person’s take on it made sense to me too because they care about the patients and I didn’t think that they were saying they’re opposed to it because they didn’t want you to get better. I think they were cautious. But I think everybody had to look at it on their own and do their own research and go with what they felt. [Helene, 6 years]

I actually had to bring copies of all my paperwork from both neurologists when I went to Egypt and I read what was said. [My neurologist] said “she is informed of all the pros and cons and potential risks and, while I can’t support her decision, I certainly acknowledge she has the right to make that decision and that she’s informed and, you know, all the power to her, make your own decision.” [Danielle, unknown years]

Another participant argued that neurologists were undeserving of scorn just for being skeptical about the new theory, because it should not discount the fact that they have been consistently working and researching towards finding better therapies for their patients for years.

### Key informant interviews

All key informants, notably all neurologists and clinicians at MS clinics, reported that the year that followed the media releases about Dr. Zamboni’s research was the one of the most difficult that they experienced in their professional lives in terms of doctor-patient interactions. They commonly noted that the first media releases framed the CCSVI hypothesis and venous angioplasty as a disease breakthrough that could signal the discovery of a cause and a cure for MS. They agreed that the stories contained limited evidence and lacked a more critical – and thus balanced – perspective. Due to limited evidence supporting CCSVI, neurologists said they were compelled to take a cautious and critical approach and preferred to ‘wait and see’ what future research would hold. Yet it was with these first news stories in mind (and ensuing social media advocacy) that PwMS came to their doctors desperate for information and possibly for help with accessing the new therapy. And so it fell to the PwMS’ usual MS clinicians to attempt to answer those questions with the very little information that they had – since they were essentially hearing about new developments at the same time as the public – and try to provide the more balanced evaluation of the issues. Furthermore, they also had to then inform their patients that testing and treatment for CCSVI would not be available in Canada until there was clear evidence to validate the hypothesis – something that might take years to complete or might never even happen.

#### Hostility towards MS clinicians

As key informants noted, this more critical stance was not very well received by many PwMS and their families. They saw that while many PwMS accepted the more cautious approach recommended by their neurologists, others saw it as dismissiveness or inappropriate ‘gatekeeping’ of patients from a potentially revolutionary treatment. MS clinicians felt that they became the “demonized” specialty and many neurologists reported being on the receiving end of considerable hostility, and some even reported receiving death threats.

The media told them there was a treatment that would help them. But they couldn’t have it. And it was primarily the people who cared for them that were creating the barrier for them to get this treatment. So during that time we were threatened. Hate mail was sent. Death threats were sent to physicians. [Non-neurologist MS clinician]

The MS public and the general public weren’t ready for that critical appraisal of it. It was almost too much like cold-water-in-the-face, I suppose, to people. Initially, I had some very angry people who demanded the treatment and demanded that I make it happen. [Neurologist]

It was remarkable how many patients were very, very angry at neurology, yeah. [Non-neurologist MS clinician]

#### MS clinicians’ perspectives of their discussions with PwMS about CCSVI and venous angioplasty

All of the MS clinicians interviewed for this study indicated that they had done their best to share what information they could about CCSVI or had directed patients to where they might be able to find reliable information. They all described how they had told PwMS that there was little evidence to support the hypothesis of a link between CCSVI and MS, and that the best course that they could recommend at the time was to wait and see what future research will show. As for venous angioplasty, they generally acknowledged to their patients that there seemed to be some anecdotal indications of some symptom relief, but there was no evidence yet in terms of the actual efficacy of therapy, of success rates (including long term versus short term), and of the safety of using angioplasty – a treatment developed for arteries – on jugular veins. In addressing PwMS’ wishes to access therapy, clinicians often claimed that while they could not recommend or endorse venous angioplasty, they recognized the right of their patients to make their own decisions. Most said that they had indicated support for their patients’ decision making by sharing the current state of benefits and risks and by continuing their care afterwards if they chose to seek treatment. As clinicians pointed out, some PwMS received the more critical appraisal of CCSVI positively, while others did not, and some PwMS said they were going to go for treatment anyway.

I would give them the information that I had [about CCSVI], which developed as research developed in the field. There are some people who will hear this information and say “oh my goodness, this is not for me, this is so experimental, I kinda want to see where this goes.” But there are some people who say to me “you know what, I kinda want to try it anyways.” I don’t think that’s wrong; you’re just trying to do the best that you can for your disease and I think that if you hear this information and you still want to do it, well then you’ve educated yourself as much as you can about the situation, make sure you try and go to a place that has a good track record and things. So I found that most people, when I would say that to them beforehand, you know, we’re going to discuss CCSVI, you’re going to hear some of my views, but ultimately it comes down to your decision, everybody responded to that in the end, almost universally. [Neurologist]

Some key informants concurred with what the focus group participants reported above that they too had heard that some neurologists were refusing to even discuss CCSVI-related issues at all. However, rather than attribute the lack of discussion to outright dismissal of patient concerns, one key informant noted that one possible reason for that was due to the practical rules and ethics that a neurologist may feel bound to, including time constraints but also the need to attend to the ongoing disease and symptom management that is necessary. CCSVI was an unproven theory and discussing it took time away from other pressing matters.

They have a very short period of time that they have to see patients. And often these patients would come and didn’t want to talk about what was going on that needed attending, that the physician was worried about. They [patients] went straight into CCSVI. And in a 30-minute appointment [CCSVI took up all the time]. And then the patient left without addressing any of their concerns about frequent falls, about UTIs that could kill them if they developed urosepsis, aspiration issues. So the things that they were trained to assess for and treat the patient wouldn’t address, and so they had to be very structured, the conversation, in a very organized manner. [Non-neurologist MS clinician]

Nevertheless, if a PwMS saw that their neurologist did not want to discuss CCSVI, most key informants conceded that patients could understandably interpret such treatment as a dismissal of their concerns – an egregious one if the PwMS sees the potential for a cure or more effective treatment at stake.

#### Communication breakdowns and other fallout

Whether by refusing to talk about CCSVI, informing about lack of evidence and uncertainties in risks and benefits as well as the lack of domestic access to venous angioplasty, or recommending against accessing venous angioplasty as a medical tourist, all key informants had reported that they too had experienced or heard about particular consequences when PwMS did not hear what they wanted to hear. Commonly, they noted that PwMS would often not tell their neurologist that they were going out of country to receive venous angioplasty, and their doctor would only find out after the fact, if they found out at all. They speculated that given the cautious and more critical stance neurologists had about CCSVI, PwMS did not want to be embarrassed in admitting that they went (especially if the therapy was unsuccessful, or less successful than anticipated), or did not want to risk the (perceived) anticipated ire of their clinician, who could potentially castigate them for their decisions (whether before or after they decided to access treatment). Other clinicians reported that they knew of or had patients who no longer went to their neurologist, or who switched to one that they perceived as more open-minded:

So no, they didn't advertise their intent before and I think in some ways they knew that the neurologist thought that this was a load of nonsense and so they didn't quite believe the neurologist so they thought they were going to do it anyway. And some of them, I almost had a sense that they slunk away to get it done, you know. [Non-neurologist MS clinician]

A lot of people, they really did sever their relationship with their neurologist. [Neurologist]

I know a lot of patients who went and had it done who claim they never told their neurologist. Because they *assumed* how their neurologist would act, right? Or react. Probably they wouldn’t, but they kept hearing stories from other people that oh, my neurologist got mad at me. When we were given the opportunity, to try to show the pros and cons, I think partly what happened is that people who really wanted to have it done stopped talking to us. Because they didn’t like our perspective that we needed to wait for the research, so they essentially stopped and started talking to people who were providing the other side of the story. [MS advocacy organization]

Many key informants lamented the breakdown of communication and relationships between PwMS and their clinicians, given the crucial role that the doctor-patient relationship plays in managing a chronic and progressively debilitating disease like MS. Even some seemingly ideal doctor-patient relationships did not withstand the wedge that the issue thrust between them.

Like I felt patients who had very strong, really positive trust and clinical relationships with their neurologist who didn't trust their neurologist anymore, because the neurologist was telling them “don't go for CCSVI, we don't have enough information.” [Non-neurologist MS clinician]

However, following the early period of hype, media intensity, and controversy about CCSVI, clinicians noted that some of the interest and discussion of the therapy had gradually died down, along with some of the animosity towards neurology. Many PwMS had also since been returning to their usual routines of care. Nevertheless, that time was not without its consequences, both short and long-term. Some communication breakdowns may take time to repair, while some may never recover. Key informants noted that many MS clinicians and nurses had seriously contemplated leaving the field of MS care, due to the intense pressure and negativity that they had endured. Some neurologists also indicated that since the CCSVI debate cast such public ire on neurologists, few neurologists are now looking to specialize in MS.

And I was really quite shocked by the negativity toward neurologists, I have to say. And it has had a negative impact, because we have not had anybody new… none of our grads have gone into MS since. [Neurologist]

#### Bedside manners – empathy, respect, and hope

Inevitably, some PwMS would not agree with the more critical stance towards the CCSVI hypothesis. While all clinicians agreed that they had done their best with what little information they had in providing a more critical assessment of the unproven hypothesis, a subset of those interviewed indicated that the crucial aspect in getting this information across to PwMS was not solely *what* was being said, but also *how* one was saying it. Two key informants used the term of having good “bedside manners” to describe what others also recounted as empathy, respect, and a willingness to acknowledge the perspectives of PwMS. This, they maintained, was the best way to increase the odds that a breakdown of communication did not occur and that a positive relationship could be continued no matter what their patient decided to do. Many key informants indicated that they recognized that PwMS were a particularly vulnerable population, frustrated by the lack of knowledge over a cause or cure for their disease, and facing an uncertain yet progressive disease. Against this prognosis, PwMS are understandably desperate for, as one interviewee said, “a new ray of hope.”

If people feel that you have already made up your mind and you’re not as impartial as you think to the new notions, they will not like you. And especially when you just erase their hope, and you offer nothing else. Put yourself in the patient’s shoes -you have an untreatable disease, and you’re looking desperately for hope, and some new things emerge, a little bit of hope, very little, and the doctor says no, it doesn’t work, it doesn’t work. Okay, what do you have to offer instead? [Neurologist]

A clinician who has a rigid mind with the blinders on, no it doesn’t work, no it doesn’t-, okay it doesn’t work, but say it differently so people can digest it and can realize that you’re on their side, but you don’t recommend it. [If] you have this oppositional strategy, in practice, that’s the source of all the problems. [Neurologist]

Participants agreed that to be dismissive of PwMS’ interests and perspectives towards CCSVI was to take away their hope and potentially signal an adversarial posture. Moreover, appreciating the sense of hope that the unproven and risky therapy offered was also a way to understand the high risk tolerance that PwMS exhibited when they opted for liberation therapy even though they had been apprised of its uncertain benefits and potential for risks.

Participants noted that many PwMS likely would not have switched or severed their relationship with their neurologist if they had been treated in a more empathetic fashion. Listening to their patients and being open and willing to engage with the concerns of PwMS were keys to maintaining a good relationship. Even if PwMS chose to go against their doctor’s recommendation, they would feel that at least they had been heard, had their concerns validated, and know that they would still have the support of their healthcare provider if they chose to be a medical tourist. One doctor noted that approaching their patients in this way actually saved time during appointments, as it prevented more adversarial postures that could grind conversations to a halt or a stalemate, and it facilitated outcomes that were more agreeable to all if PwMS were set on seeking treatment.

So I think it has to do with listening. And being open. And sometimes if they want to take you in a particular direction, it might not take very long to talk about that direction, but to just have an openness of thought, that actually it saves time in the appointment overall….But it’s so easy to do. […] The trick is to not spend a lot of time with people but to make them think that they’ve spent a lot of time with you. I think that you can spend a very little amount of time with a person yet make it really count in some way or another, but it has to be real. It’s about validating, it’s about empathizing. And all it takes is a simple sentence of “yeah, I can see how you would see it that way” [Neurologist].

Several clinicians added that increased training should be given to MS physicians to help them to “put yourself in the patient’s shoes” to more sensitively navigate difficult conversations they will inevitably have with their patients. On the other hand, some key informants also hoped that in situations such as the CCSVI debates, some PwMS and their advocates also needed to reciprocate those sentiments of empathy and openness in kind, and that the hostility that existed towards neurologists was not necessary or helpful – as understandable as it may have been.

#### Conspiracy theories and turf wars

Most key informants also mentioned that they had heard about particular conspiratorial theories that purportedly explained neurologists’ skepticism or lack of endorsement of CCSVI. Echoing the focus group participants, the most common suspicion they heard concerned perceptions about neurologists’ relationships with pharmaceutical companies and that there was too much money being made by “Big Pharma” from DMTs. They also heard related criticism that livelihoods would be lost if CCSVI was indeed a cure, and hence any perceived skepticism was actually viewed as professional self-preservation.

Like a conspiracy theory that, you know, the drug companies were fuelling this [skepticism of CCSVI] and all the disease modifying companies don't want people going through the surgery because it will take away the money from their drug. [Non-neurologist MS clinician]

[Many patients] regarded many of us as withholding a therapy that would make them better and therefore not need our services and so therefore we were sort of protecting our livelihoods and maybe we were being paid off by pharma and so that’s why we weren’t offering those therapies. [Neurologist]

One neurologist countered such claims by stating that MS-related work funds so little of their practice that it was impossible that pharmaceutical companies or any financial benefits were influencing their lack of immediate advocacy for CCSVI.

It was so ironic. They’re saying well, MS neurologists are making too much money treating MS, and I thought, you know what, MS is my charity work; you know, I have another half of my practice – it funds my practice. MS does not pay, you know, you specialize in MS for different reasons....that kind of got lost. You’re either for it or against it [CCSVI and liberation therapy], that if you weren’t lobbying for immediate access, you were against it. [Neurologist]

Clinicians who were interviewed also flatly denied any suspicions that there was a kind of ‘turf war’ between neurologists and the specialties associated with CCSVI – radiologists and vascular surgeons. Key informants repeatedly claimed that MS care is multidisciplinary and that neurology often collaborates with other specialties, and did so again when learning about CCSVI.

I disagree with that 100 percent [that there was a turf war]. There was some mutual discussion within the vascular arena. There were physicians who we brought in to give us some help with this. There was a lot of collaborative work that went on during this crisis so that neurologists could help understand, the vascular people could help understand, that if they looked at techniques how would this work. They really worked collaboratively. They really did try and help each other out because both had pressure from different world views of medicine. [Non-neurologist MS clinician]

#### CCSVI-induced changes in MS advocacy and communication

Some key informants shared that they thought that amidst all the challenges that the CCSVI debates created between them and PwMS, the situation had also stimulated some necessary changes and realizations. They noted that PwMS and their advocates had found a new voice for themselves and that the relationship dynamics between PwMS and clinicians had likely been changed irrevocably – changes that may be difficult, but could also be for the greater good. Many clinicians agreed that, in the end, open and collaborative communication with PwMS needs to be improved.

It galvanized a whole group of people to act out in a certain kind of way and advocate for their illness, which I think has been good in the end. And it’s made neurologists approach their patients differently. I think that it should be a collaborative discussion; I think it should be a discussion over how much do you know about this, what don’t you know about it. You know, bring stuff in from the internet and show it to me and if I don’t know about it we’ll look at it together on the internet and we’ll try and figure it out together and I will just be honest with what I know and don’t know. [Neurologist]

And I hope that [what] comes out of this whole issue in Canada, is how should we – as the scientific, academic, clinical community – communicate better with patients. Because, you know, let’s say... there’s different scenarios that can come out of the CCSVI thing. Let’s say the predicted scenario, is that it ends up just being noise. Just a fad. It was weak science and it can’t be reproduced. So let’s say if that is the scenario, are people going to remember this five years from now? All the money that was spent, all the emotions that were hurt and bruised? Or is it just going to be forgotten and something else is going to go through the same process again? The other is I think it’s created dialogue; dialogue that hasn’t happened before. It’s made people more aware of the power of the internet, the social community, how well aware MS patients are of the issues, how well educated they are. I think it’s forcing us clinicians to be better communicators. We still... We gotta listen to our patients. It’s tough sometimes, right? Like I don’t want to spend my whole clinic talking about CCSVI. I’d rather talk about some other things. But it’s forced dialogue. [Neurologist]

As described in the above comments, there are clearly lessons that stakeholders want to draw from this experience. The controversy and its consequences forced some key informants to reflect on their experiences and use them as a guide to improve doctor-patient communication.

## Discussion

Could a clinical dynamic guided by principles of SDM still prove amenable to a highly controversial context like CCSVI? The spectrum of perspectives found in our results point to both the potential and limitations of SDM when faced with a controversial new hypothesis and a public debate being played out in real time. The neurologists we interviewed all described their CCSVI-related discussions with PwMS in ways that aligned closely with SDM – although only one key informant actually used that term to describe their approach and several others used the term “informed decision making” (and both have been used synonymously in the MS context [19]). They all self-reported that they had openly discussed the state of limited evidence about CCSVI and risks and benefits of venous angioplasty, encouraged patient inquiry, and with each of these factors in mind felt compelled to recommend against seeking treatment of this kind or waiting for more evidence. Further, they all noted that they left the ultimate decision to seek venous angioplasty to their patients and that they would continue to support them if they did so. Key informant clinicians indicated that this approach was crucial in maintaining their responsibilities to the evidence as well as to the good care of their patients. Of course, it may be possible that a clinician’s self-assessment of their own actions may tend towards the positive, and these more benign perceptions should be contrasted with the widespread negative characterizations that PwMS participating in this study shared regarding their interactions with their own clinicians about CCSVI. Outside of the MS context, research has shown that despite doctors’ claims to be adhering to SDM, surveys of patients have claimed otherwise [46]. After reviewing our results, it is not possible to make a judgment about whether the neurologists we interviewed simply over-represented those who exhibited good ‘bedside manners’ or were assessing their own practices in a more favorable light. Nevertheless, it should be taken as a positive sign that all MS clinicians we interviewed described their experiences in ways that modeled SDM principles and believed that it was due to this patient-centered approach that they had many positive or satisfactory responses from their patients, and minimized as best as possible the potential for a breakdown of trust and communication.

Some PwMS participating in this study also shared positive experiences they had with their doctors when they wanted to discuss CCSVI and venous angioplasty. They appreciated their neurologists’ efforts to be open and hear their concerns, to provide them with what information that they could, and to support them even if they sought out liberation therapy. These perspectives lend further support to the value of the SDM approach when discussing even novel, uncertain, or controversial therapies. These participants were freely able to inquire about their concerns, feel reassured that their opinions and values were validated and respected, and could be certain that they could maintain the continuity of specialist care that is crucial to optimal MS disease management [47, 48]. These positive outcomes are valuable even in light of the fact that not all facets of SDM could be present in their ideal form (i.e. where there is a much stronger evidence base (at the time), the availability of patient decision-aids, etc). Even though the evidence base was lacking on which to make decisions about CCSVI and its testing and treatment were not accessible therapy options in Canada, the crucial point is that in many cases even when it is only possible to affirm the values of the patient, that alone may be all that is necessary for relationships to be maintained and prevent breakdowns of communication.

Although these positive results are encouraging, we also found many examples where the perceptions of PwMS of their clinical discussions of CCSVI were decidedly negative, and these too can be analyzed on the basis of the SDM model. Further, they illuminate the rationale behind some of the unfortunate consequences of those perceptions, whether being communication breakdowns, loss of trust (and having to rebuild it), severing of relationships, independently altering/ceasing existing treatment courses, or hiding decisions to seek venous angioplasty.

Some PwMS reported that their neurologist had refused to talk about CCSVI and saw this as a trend associated with all neurologists – or the field of neurology – more generally. This left focus group participants with many of their questions unanswered and the perception that their neurologist was preemptively dismissing their pressing concerns amidst a time of widespread public controversy and interest. While all neurologists in this study maintained that they had not done so, our key informants also corroborated that they too had heard of neurologists who had refused to discuss CCSVI with their patients. These reports stand in stark contrast to SDM’s promotion of an open and two-way exchange between physicians and patients [19]. Of course, SDM is premised on the sufficient exchange of risk and benefit information about treatment options – which for CCSVI and liberation therapy was insufficient at the time. Nevertheless, simply avoiding the topic – however valid or practical the reasons on the part of the clinicians – was viewed by MS participants as a significant rebuff of their concerns and could feed and nurture existing adversarial narratives in the media or already existing discontent that PwMS had with their neurologist. Refusal to engage with their concerns were the key factors behind PwMS making drastic decisions to quit their neurologist or heed the advice of those more likely to listen to them or to be supportive of CCSVI. Thus, even if a therapeutic option does not fit the profile of an established DMT or deviates from matters of importance from a clinician’s perspective, to prevent a breakdown of communication, care must be taken not to be insensitive or indifferent to discussing matters of importance from the perspectives of PwMS.

Not all neurologists were dismissive of CCSVI if PwMS wanted to discuss it. All of the key informant clinicians interviewed indicated that they engaged in related dialogues with their patients and shared information as they could. As noted above, many focus group participants responded positively to this more critical approach, but as was also found in our results, it was not what some wanted to hear and could also engender a breakdown in communication. As a result, some clinicians noted that they still had patients who sought venous angioplasty and who did not tell them about it. Some PwMS also shared that because once they knew (or perceived to know) where their neurologist stood on the issue, they saw no further point in belaboring it or in discussing their private decision to seek liberation therapy. While being open and collegial about patient concerns fulfills an aspect of SDM and can be sufficient for some PwMS in making a decision based on what is or is not known about a novel hypothesis, it may not satisfy others who approach their condition differently and may have differing preferences or levels of risk tolerance – tolerances that reflect the length of time they have had MS or their level of disease progression [21, 49].

This last point bridges onto another facet of SDM that is also of critical importance for health decision making: the opportunity for the patient to share their values [19]. Indeed, patients bring preferences and personal values to many kinds of clinical contexts, but they likely obtain greater salience – and emotional gravitas – in the case of a progressive disease like MS or other physically debilitating chronic diseases with few therapeutic options. SDM involves conversations that seek to balance the evidence of known benefits and risks with a patient’s preferences and values – that is, what patients want to achieve and how much risk they want to tolerate in turn [21, 50]. For the case of CCSVI, it was not just an unproven hypothesis that PwMS wanted to discuss with their physicians, but what it also represented – hope [51, 52]. Indeed many focus group participants spoke in emotionally laden terms while describing the hypothesis as finally finding a “ray of hope,” where historically none have existed – let alone been offered. Participants processed information about CCSVI in such an intensely emotional way that it may have drowned out or overrode more critical or analytical rationale [53–55]. Even if neurologists offered a more balanced illustration of the evidence, they could still overlook what even a weak hypothesis meant to PwMS suffering from a progressive disease with (hitherto) no known cause or cure [41]. Facing this prognosis and given even a remote chance of a better life, many PwMS were willing to forgo their neurologists recommendations and take the risk. Notably, and in keeping with recent research [49, 56], most focus group participants who sought out venous angioplasty had more progressive forms of the disease and consequently felt they had less to lose and potentially the most to (hopefully) gain by taking on the increased risk of an unknown therapy. Furthermore, any frustrations of their hope or desire for more information would likely come to be directed at those who seemed to stand in the way – their neurologists. PwMS with an unsatisfactory relationship with their neurologist would have seen this as just another disappointment among others.

As noted by key informant clinicians, PwMS responded best to their more critical discussions when doctors invited and validated the values and emotions (hope, desperation, etc.) of their patients. Therefore, clinicians who ignore or dismiss the emotional cues (e.g. hope, worry, desperation) of PwMS do so at the risk of missing a crucial part of how patients understand a new hypothesis as well as their disease [8], and as illustrated in our results, the unfortunate possible consequences when patient values or preferences are ignored. Many key informant neurologists described how they tried to put themselves “in their patients’ shoes” or assume a more empathetic “bedside manner” in their discussions of CCSVI. They noted that assuming this kind of posture was a key reason why their relationships with many of their patients were able to endure through such a stressful time and that this element was likely missing for PwMS who severed their connections with their neurologist or lost trust. Some focus group participants also reported a desire for their clinician to exhibit better “bedside manners,” meaning better communication skills and respect for the hopes and values of PwMS. Such an approach is in keeping with SDM and its commitment to mutual trust, creating shared understandings, and respect for more autonomous patient decision making – even when that decision may be contrary to the clinical recommendation presented. This highlights the importance of actively eliciting the values of the patient, and validating their emotions in addition to presenting the scientifically-based perspective of an unproven hypothesis. Indeed, it may well be better to consider addressing the former with PwMS even before presenting the latter – in fact, it may be the only facet of SDM that is possible in a situation where a clinician may themselves not even know what evidence exists. Admittedly, if a clinician avoids discussion of a topic because they too lack knowledge about it, it could be potentially perceived as a rebuff of patient concerns as well. In other words, before an MS clinician tells their patients what they know (or what is known or not known), they should first identify with how PwMS feels and connect with those values, which may in turn create a more respectful discussion of the state of the evidence [57]. This may form a basis of trust that may make more biomedical discussions of practice or evidence more acceptable to PwMS [58–60], and further build a stronger foundation that can help all parties weather these inevitable challenges. As was suggested by some key informant neurologists, further training in such approaches of good “bedside manners” may be necessary for MS clinicians when facing such highly emotional contexts.

Nonetheless, even with greater emphasis on good “bedside manners” or with heeding the call of SDM for greater integration of exchange of information and values between doctors and patients, there still existed the potential for misattributions of intentions that flowed from both directions – from perspectives of clinicians and PwMS. Of course, there were instances where PwMS and their neurologists found their opinions to be well-received and acknowledged by each other. However, in many other instances neurologists may not have believed that they were being unduly negative or dismissive by giving little time or a more balanced view to CCSVI, but rather that they were just exhibiting the necessary caution for an unproven hypothesis. On the other hand, PwMS may believe that their inquiries about CCSVI are of critical relevance to their care, but may indeed misattribute clinical caution as undue negativity or dismissal of concerns. Regardless of the intention of either side, PwMS can be left feeling as though they are not being heard, and clinicians can be left feeling unfairly characterized as “villains” or as indifferent. These kinds of disconnects may prove a foil at times even for the SDM approach, especially in this particularly media-driven and emotionally charged context where there were spectrums of perspectives and expectations on both the patients’ and clinicians’ sides. In the end both sides need to be effective communicators with each other; SDM may be the leading model for minimizing breakdowns of communication between PwMS and their clinicians. At the same time, the dialogical approach of SDM is always subject to prevailing expectations and biases of either side, which may always hold the potential for a misalignment of intentions, perceptions, and understandings. Furthermore, while SDM is modeled on participants co-creating mutually satisfying outcomes, the context of CCSVI shows that it may not be so straightforward a goal. In fact, in situations where a person believes that their last hope for improving their quality of life is at stake, the satisfaction of a patient’s desired outcome may move to the forefront from the patients’ perspective. This case study therefore also sheds light on these particular tensions – among others noted above – and the challenges they can pose to the SDM type of approach in specific clinical contexts. Such tensions are sure to come to the fore again in future scenarios where a ‘breakthrough’ hypothesis (whether for MS or other diseases) forces stakeholders to navigate unique and complex decision making processes when the stakes are high but the evidence is lacking or uncertain.

## Limitations

There are some specific limitations to this research worth noting. First, we did not conduct paired interviews with the health professionals of our focus group participants of PwMS but rather reported the general statements made by PwMS in only one city as compared to the reflections of other key informant participants (neurologists, other health professionals, advocacy organizations) from across Canada. Second, the general tendency to present oneself in the best possible light is a characteristic concern of any research that relies on self-report; but it is even potentially more relevant when examining statements within a highly controversial public debate such that participants may not have accurately represented their actions. However, the similarity of the groups in the reported challenges in how PwMS discussed CCSVI with their clinicians (whether from their own experiences or others they had heard about) do overall substantiate each other’s testimonies. Third, while this case study focuses particularly on the doctor-patient discussion of CCSVI, the views that PwMS had of those discussions were not in a relational vacuum, but were part of a context of care that may or may not have preceded the emergence of the CCSVI debate. Thus, PwMS interpreted their discussions (or lack thereof) within the context of their overall care of their neurologist such that particular broader issues may have influenced participant responses to the research questions that were central to this study. Fourth, it is possible that the experiences reported here may not be generalizable to other countries given how the CCSVI/liberation therapy issue was handled by our news media as well as by the shifting policy responses of different levels of government. Last, there was a considerable time-lag between when the research was carried out (especially for focus groups held in 2012) and the study’s publication. Nevertheless, the results of the study are still instructive for the inevitable ‘next time’.

## Conclusion

SDM was initially recommended for MS doctor-patient relationships to improve communication and decision making with respect to prevailing MS therapies, or DMTs. However, when a controversial hypothesis arises, it can put those relationships and principles of SDM to the test and force new or latent challenges to the surface. Within the context of this research, even though the CCSVI debate was not the ideal setting for SDM, when its core elements of dialogue promotion and values acknowledgement were present it decreased the likelihood of breakdowns of communication. Instructively, there exist patient decision aids for more general health screening, testing, and treatment services for improving decision-making in the clinical context. These decision aids also emphasize the importance of clarifying patient values for medical “grey areas” where the status of evidence is conflicting, or at least not entirely conclusive [50].

The CCSVI debates exposed and at times amplified communication problems between PwMS and their neurologists. While some of those relationships coped well enough, many others were left broken or bruised with struggles that continue to this day [61]. It also gave PwMS an opportunity to gain more of a voice for themselves in the management of their disease. To fix existing problems and to accommodate the values of PwMS as well as the recommendations of their clinician – even in the context of a new and controversial hypothesis – incorporating the principles of SDM even into uncharted and unanticipated areas of MS disease management can be part of the ongoing efforts toward making necessary improvements in MS doctor-patient communication and care.

## Additional files

Additional file 1: Detailed Methods for “Caught in a no-win situation: Discussions about CCSVI between persons with multiple sclerosis and their neurologists – a qualitative study”. This document provides a much more detailed of the study’s methods, with particular close attention to the process of data collection and analysis. (DOCX 40 kb)

Additional file 2: CCSVI Focus Group Interview Guide. This document contains the semi-structured focus group instrument that guided discussions with people with MS regarding the CCSVI issue. (DOCX 35 kb)

Additional file 3: Key Informant (MS) Interview Questions. This document contains the semi-structured key informant interview guide used for clinicians, researchers, health policy makers, and advocacy organizations. Various individual tailoring for specific groups are represented in this document, but as interviews progressed, further tailoring was undertaken as most relevant for that key informant stakeholder group. (DOCX 26 kb)

Additional file 4: Consolidated criteria for reporting qualitative research (COREQ) – Checklist. This document contains a checklist used when reporting qualitative research to ensure greater transparency around data collection, analysis, and reporting processes. (DOCX 22 kb)

## Abbreviations

CCSVI: Chronic Cerebrospinal Venous Insufficiency
DMT: Disease modifying therapy
MS: Multiple sclerosis
PwMS: Persons with multiple sclerosis
RCT: Randomized control trial
SDM: Shared decision making
