# Supplementary material for: Caught in a no-win situation: discussions about CCSVI between persons with multiple sclerosis and their neurologists – a qualitative study
Source: BMC Neurol. 2017 Sep 7;17:176. doi: 10.1186/s12883-017-0954-7 (PMC5590111; doi:10.1186/s12883-017-0954-7)
Supplement: Supplementary file 1 — Detailed Methods for “Caught in a no-win situation: Discussions about CCSVI between persons with multiple sclerosis and their neurologists – a qualitative study”. This document provides a much more detailed of the study’s methods, with particular close attention to the process of data collection and analysis. (DOCX 40 kb) [file 12883_2017_954_MOESM1_ESM.docx]

**Additional File 1: Detailed Methods for “Caught in a no-win situation: Discussions about CCSVI between persons with multiple sclerosis and their neurologists – a qualitative study”**

## Study Overview

This research was conducted as part of a broad mixed-methods study examining the management of uncertainty in decision making. The initial project was focused on cancer control policy in Canada that is funded by a grant held by author SMD from the Canadian Cancer Society Research Institute entitled “Advancing quality in cancer control and cancer system performance in the face of uncertainty” (grant #700589). At the time that project was funded, the CCSVI hypothesis was gaining media attention and prompted the lead author to secure funding from the Multiple Sclerosis Society of Canada to fund the MS portion of the case study (EGID #1261) as part of this larger program of research. Authors SMD and RAM are the co-principal investigators of the MS study. In addition to the CCSVI and venous angioplasty case study for people with multiple sclerosis, the larger program of study is examining four cancer control scenarios that are characterized by varying kinds and levels of uncertainty: (1) mammography screening in women aged 40 to 49 years, (2) prostate-specific antigen (PSA) screening, (3) the approval and funding of new cancer drugs, and (4) school-based HPV vaccination programs.

The overall study has three broad components: (1) an integrated review of empirical research (quantitative and qualitative) and non-empirical works (e.g., theory, conceptual frameworks, commentary, etc.) about uncertainty in health policy decision making; (2) qualitative research involving focus groups with members of the public (or in the case of MS, people with MS) and key informant interviews with senior officials who make decisions about or influence policy in various organizations in the Canadian health system, and in the case of MS, we also included physician specialists, researchers, and MS advocates; and (3) quantitative empirical research leading to the development of a practical tool to help policymakers assess and manage uncertainty in their analyses, and ultimately make better and more transparent health policy decisions. This paper deals with component 2, in the exclusive context of MS.

## Ethics

The research protocol, consent forms, and data collection instruments received ethics approval from the University of Manitoba’s Health Research Ethics Board (H2012:184).

## Focus group Participants and Recruitment

In June, 2012 we held seven different focus groups containing a total of 69 people living with MS. Each group was made up of people with different types of MS (relapsing-remitting and primary or secondary progressive) who have lived with their diagnoses for different lengths of time. The breakdown was: 26 progressive; 30 relapsing-remitting, plus 13 mixed in our pilot test group. Length of time people had lived with MS were: less than five years, 10 to 19 years, and 20-plus years. The make-up of the groups was as follows (with some mixes depending on individuals’ schedules):

| **Date** | **Number of participants** | **Type of MS** | **Years Diagnosed** |
| --- | --- | --- | --- |
| **June 11**  **(pilot group)** | **13** | **Mixed** | **Mixed** |
| June 25 (morning) | 11 | Progressive | 20+ |
| June 25 (afternoon) | 8 | Relapsing-Remitting | Less than 5 |
| June 26 (morning) | 5 | Progressive | 10 - 19 |
| June 26 (afternoon) | 11 | Relapsing-Remitting | 10 - 19 |
| June 27 | 11 | Relapsing-Remitting | 20+ |
| June 28 | 10 | Progressive | Less than 5 |

Participants were recruited through a research coordinator affiliated with the Winnipeg Multiple Sclerosis Clinic via a registry of MS patients kept at the MS Clinic (ethics approval H2011:101) as per its established protocols. This registry comprises people with MS who have provided consent to be contacted by a research coordinator at the MS Clinic to participate in other research studies. This research coordinator recruited participants, shared study information sheets and consent forms, and obtained consent to have their names shared with the primary study team (SMD and a research coordinator) that would be leading the focus groups. However, it was the study lead (SMD) that formally consented participants into this study for the focus groups. No clinicians affiliated with the study (or otherwise involved in MS Clinic care activities) were involved in the recruitment or consent process. Decisions to participate were entirely voluntary. An honorarium of $60 was provided to compensate people for their time, along with parking costs and some light refreshments.

**Key informant Participants and Recruitment**

Potential participants were identified by study team members and from policy experts and clinicians involved directly in, or in collaboration with our partners’ organizations (e.g. MS Society of Canada, provincial health officials, the Canadian Network of MS Clinics). Initial telephone and/or email contacts were made by the project lead (SMD) and/or project coordinator.

## Data Collection

Established protocols for conducting focus groups were followed [[1-3](#_ENREF_1)]. The focus group interview guide was pilot tested in one focus group that included both relapsing-remitting and progressive (primary and secondary) MS. The final version of the instruments did not include any substantial revisions, so the data from the pilot focus group was pooled with the remaining focus groups for analysis.

The focus group discussions were guided by an interview guide (see Additional File 2) that began with open-ended questions about people’s first thoughts about MS and then about CCSVI, people’s information seeking preferences, discussions of whether people traveled to have venous angioplasty performed, including their experiences, how they arrived at the decision (level of discussions with different types of healthcare providers (or not), and so forth, before introducing more broadly the challenge the CCSVI hypothesis had created on the Canadian policy environment, including positions that were taken at the federal and provincial levels, as well as that of the MS Society of Canada. To expand on this subject further, we opened up the conversation to how society generally should think about where health research dollars should be spent when scientists have not yet been able to agree on the evidence about such treatments, what provisions should be in place for these discussions, and so forth. We ended each focus group session asking what participants would like to say to health policymakers about managing MS and venous angioplasty if they were able to talk to them at the moment. There were no specific questions about shared decision making, but aspects of this, both explicitly and implicitly, were freely raised by participants as they discussed their interactions with their healthcare providers.

Key informant interviews followed a convergent interviewing process [[4](#_ENREF_4), [5](#_ENREF_5)]. An interview guide is developed (see Additional File 3), but where there are some general opening questions that the interviewer builds upon in developing the conversation around participant reflections on what worked well (or not) in particular situations. In subsequent interviews, after a participant has explored the topic more fully within the interview process, convergent interviewing, by design, creates opportunities for the interviewer to ask participants to reflect on what a different participant experienced to see if that circumstance was relevant (or not) to their situation. By so doing, data saturation is reached more quickly. However, as we were interviewing different groups of key informants, we ensured that saturation was reached for within that particular participant group.

In this particular case study, all key informants (clinicians, policy makers, researchers, advocacy organizations) were asked about their job position and responsibilities as well as about how decisions are made (at a policy or clinical level as relevant) regarding the approval of treatments for diseases like MS in Canada, asking participants to reflect on how input from different stakeholder groups (patients, clinicians, researchers) are incorporated, and what they see as the strengths and challenges of that general process. General questions also probed areas for sources of uncertainty (clinical, evidence, financial (health system impact in balancing clinical/individual vs population/societal impacts), policy level, etc. ) and how that is managed by them. However, early on in the interview conversation if a participant raised the issue of CCSVI and venous angioplasty, those aspects would be explored with them in terms of what they saw as the defining moments in the issue, what challenges they faced in trying to manage patient/public pressure, and so forth. Because of the different types of participants involved, different interview guides tailored to specific participant types (clinician, policy, advocacy, etc) were generated, with specific prompts to explore with those participants as part of the convergent interviewing process that would be most relevant to them. Consequently, the interviews needed to be led by the project lead given her years of experience in qualitative inquiry.

## Data Analysis

All focus groups were digitally audio-recorded, transcribed verbatim, audio-verified against the recordings and edited to correct any errors. All data were analyzed using NVivo9 and then 10^TM^ qualitative data management software. The project lead, SMD, is an expert in qualitative methods and a trainer for using NVivo ^TM^. Her typical strategy for coding data involves doing a first level of coding of surface level descriptive content. What this entails is to code lines of text into different content categories of meaning in order to group those ‘like’ ideas within the same category across the dataset. For example, participants were asked about what risks or harms had ever been mentioned to them, or that they had read, about Liberation Therapy. Any reference that participants raised about risks would be coded in this node. This enables the researcher to examine how people discuss and contextualize notions of ‘risk’ however defined by the participants.

Because qualitative analysis does not aim to precisely quantify qualitative data (e.g. X% of lines of text across the dataset focused on Idea Y), it is common, and in fact strongly recommended, to code lines of text multiple times into relevant content categories as appropriate. For example, Bazeley [[6](#_ENREF_6), [7](#_ENREF_7)] recommends using fewer descriptive content categories or nodes, but capturing relevant aspects in terms of what is being described, e.g. is it being described positively or negatively (if that can be assessed or relevant), and so forth. The aim is to be able to put together more sophisticated questions that can be asked of the dataset through coding and matrix queries once the dataset has been initially coded.

SMD operationalizes this strategy by dividing surface descriptive content categories very broadly into *who, what, why*, and occasionally *where and when* categories as relevant. A who-type-category is designed to capture the people or organization being discussed (e.g. health care provider, family/friends, government, media, experts, etc). A what-type-category involves the subject matter of what is being described in the sentence, sentence fragment, paragraph, or conversation between a set of participants (as in the case of focus groups). A why-type-category captures more interpretive aspects within any set of text. For example, if a participant describes that she has not had much success with disease modifying drugs, finding her physician unwilling to discuss materials she brought in concerning non-standard alternatives, and expands on why she felt she needed to seek diagnostic tests to see if she had blocked veins in her neck out of country, this would be coded as follows: decision-making process (because of disease stage and relative unmet therapeutic need), physician (because she is discussing strategies she used with her doctor), information seeking and processing (because of her information searches), communication (to capture challenges with physician-patient communication), and so forth depending on the exact nature of the lines of text in question. Likewise, if someone is talking about something in a negative or positive manner, these also become coded as positive or negative. These nodes are more liberally assigned and, occasionally subjectively interpreted by the individual coder, which is why processes of reflexivity and researcher triangulation (described further below) are needed. In building on the previous example above, instances of a participant describing her concerns about her disease progression with her physician but not feeling that her physician is being sufficiently receptive to discussing her concerns, would be coded as ‘negative’. Likewise, someone under the same condition, if finding their physician, while not supportive of her seeking venous angioplasty, is at least respectful in hearing her concerns and discussing options with her, might become coded as positive.

Following this general process that structures very broadly who, what and why type categories, authors SMD, RM, and two other research staff developed a codebook by independently reviewing a sample of transcript excerpts and iteratively developing draft codes and operational definitions based on the descriptive content of the text itself. After comparing their draft coding schemes, they resolved disagreements through consensus, and eventually agreed on the final coding framework that was used to systematically code each line of transcript text into content categories. As multiple coders were required for this and a related study, four coders would test-coded a sample of transcript excerpts to establish inter-coder reliability. After they coded each transcript excerpt, NVivo’s coding comparison query was used to output Cohen’s Kappa coefficients comparing each pair of coders’ agreements and disagreements for each node. The individual Kappa scores for each node were summed and averaged in an Excel spreadsheet. The coders met to review their Kappa scores, examined the different ways each had coded nodes with particularly low Kappa scores, and collaboratively developed more precise operational definitions to be applied in test-coding the next sample transcript. Kappa scores increased as each subsequent transcript was test-coded, until coders achieved Kappa scores of 0.91, exceeding the common > 0.80 benchmark for near perfect inter-coder reliability [[8](#_ENREF_8)]. RM and three other coders subsequently coded all the data. Salient categories emerging from the surface content coding of data and included: trust, communication, health care provider, neurological, liberation therapy, CCSVI, MS decision-making factors, emotions, hope, fear, risk/risk groups, finance/monetary issues, age, family/friends, side effects/harms, uncertainty, evidence, information, living with MS, government policy, and so forth.

In addition to this, when transcripts were being audio-verified they were also verified as best as possible for voice-attribution (i.e. to identify each speaker in focus groups). Having transcripts organized by voice attribution allows for a number of different ways the data can be analyzed. First, it enables examining how an individual expressed themselves throughout the entire focus group. In a software like NVivo, it is possible to autocode all individual speakers of a focus group into a case-node. Effectively, this creates a separate node for each individual speaker where everything s/he says is coded as their name. This is important because the researcher can subsequently examine how a specific participant discussed the topic throughout the focus group, as well as assess how s/he changed responses over the course of the focus group discussion. While it is never possible to fully ‘know’ why a person changes their perspective within a focus group, training, experience, and careful reading of the text, both as an individual case node, as well as through a participant’s conversation interactions with others, can help to ascertain if someone shifted their response given the presence of a dominant speaker or an emerging ‘group think’, or if they seemed to be changing their response because they were being exposed to information that was new to them, prompting them to rethink their original position.

Second, in doing voice-attribution within a focus group dataset, it is then possible to attach specific attribute level data to each case node in the Node Classifications function of the software. Attribute level data consists of organizational or administrative data information (e.g. age, gender, income, etc), but the researcher can also assign attribute level data to a participant based on how s/he discussed aspects in the focus group. It is important to note that attribute level data is categorical data, not interpretive data, and needs to apply to the whole case (in this case the whole participant). Researcher created attribute level data would include aspects like: participant indicated they traveled to obtain venous angioplasty as a medical tourist (i.e. where listed options would include at yes, no, thinking about it, unassigned); and so forth. Attribute level data in an NVivo project, when set up properly, allows for more detailed queries to be run of how participants discussed an issue that was then coded as an interpretive node category against specific classifications/attributes for specific sets of participants. For example, a query of how pwMS living with their disease for < 5 years might discuss conversations with their physician about CCSVI compared to someone who was living with the disease for longer intervals of time (10-19 or 20+ years). These types of queries allow the researcher to isolate those participants within a larger dataset holding particular views. Moreover, the researcher, in reviewing a series of single query outputs, can conduct a deeper reading of that text to assess the meaning participants were aiming to express again other factors (e.g. outputs from other queries, how issues have been discussed in the literature, etc).

Fundamentally embedded into the surface descriptive coding process as described above, as well as involved with more interpretive reading of the text described further below, is how a software like NVivo allows the researcher to explore ideas through the development of different Memos throughout the coding process, following protocols suggested by Richards [[9](#_ENREF_9)]. As a software tool, all data imported into the project and created within the project is stored together in a single file. Developing Memos, which effectively have the same functionality as a word-processing document, allows the researcher to explore ideas as they emerge. When doing surface level descriptive content coding, a researcher needs to frequently interrupt coding to capture elements that strike them as ‘interesting’ to examine what it is about those lines of text that captured their interest. As more interpretive deep reading occurs as a person conducts different queries of their data, Memos are to be built upon and expanded by incorporating outside literature as well as how different aspects seem to be challenged by some participants, as an example.

Similar processes were followed for the analysis of key informant interview transcripts. It should be noted that with key informants – as with focus group participants – they too were not specifically asked about shared decision making. Again, aspects of shared decision making, both explicitly and implicitly and whether named (i.e. ‘shared’ or ‘informed’ decision making) or not (but were conceptually linked), were voluntarily raised by participants often in an unprompted as they discussed their interactions with their patients (or other clinicians’ interactions with their respective patients).

Consequently, research findings as reported in the associated article that assign interpretive labels regarding elements of shared decision making was never specifically coded that way at the outset. Rather, it emerges from a deeper reading of more surface descriptive coding, alongside other coding queries. As the team processes the content of the qualitative data analysis, they assess what results cohere for different papers to be developed in reporting the data. In developing any manuscript, much more detailed and interpretive analyses are undertaken. It is through processes of reflexivity and researcher triangulation [[10](#_ENREF_10)]– where research findings and emerging analysis are discussed with other team members having different disciplinary perspectives – that any emerging analysis is challenged to assess if alternative interpretations are possible. These efforts are imperative to ensure that there is not premature closure in the analysis process. Moreover, the process of constant-comparison coding also serves to challenge the analysts in their thinking to assess if there are other interpretations possible within the text.

**References**

1. Krueger R. Focus groups: A practical guide for applied research. Newbury Park: Sage Publications; 1988.

2. Morgan D, Krueger R. When to Use Focus Groups and Why. In: Morgan D, editor. Successful Focus Groups: Advancing the State of the Art*.* Newbury Park, CA: Sage Publications; 1993: p. 3-19.

3. Liamputtong P. Focus Group Methodology: Principles and Practice. Los Angeles: Sage Publications; 2011.

4. Driedger S. Convergent Interviewing. In: Coghlan D, Brydon-Miller M, editors. SAGE encyclopedia on action research. Los Angeles: Sage Publications; 2014: p. 186-187.

5. Dick B. Convergent Inteviewing: a technique for qualitative data collection. Brisbane: Interchange; 1998.

6. Bazeley P. Qualitative Data Analysis with NVivo. London: Sage Publications; 2007.

7. Bazeley P, Jackson K. Qualitative data analysis with NVivo. 2nd ed. Los Angeles: Sage Publications; 2013.

8. Neuendorf KA. The Content Analysis Guidebook. Thousand Oaks, California: Sage; 2002.

9. Richards L. Handling qualitative data: A practical guide. 3rd ed. Los Angeles: Sage Publications; 2015.

10. Patton MQ. Qualitative Research & Evaluation Methods. 3rd ed. Thousand Oaks, CA: Sage Publications; 2002.
