# Supplementary material for: Caught in a no-win situation: discussions about CCSVI between persons with multiple sclerosis and their neurologists – a qualitative study
Source: BMC Neurol. 2017 Sep 7;17:176. doi: 10.1186/s12883-017-0954-7 (PMC5590111; doi:10.1186/s12883-017-0954-7)
Supplement: Supplementary file 2 — CCSVI Focus Group Interview Guide. This document contains the semi-structured focus group instrument that guided discussions with people with MS regarding the CCSVI issue. (DOCX 35 kb) [file 12883_2017_954_MOESM2_ESM.docx]

**Focus Group Interview Guide**

**General Opening Questions**

Please state your first name and tell us how long you have lived here.

Off the top of your head, please write down what the first thing that comes to mind when you think about your MS?

What comes to mind when you think about Liberation Therapy also known as venous angioplasty used to treat CCSVI (Chronic Cerebrospinal Venous Insufficiency)? Do not worry if you do not know what Liberation Therapy or CCSVI is. If you don’t know what it is, just write that on the paper.

Okay. Let’s go around the table to hear people’s responses, and don’t be shy if your answer has already been read out.

Are there things about multiple sclerosis you’re not sure about?

*Probes:*

What causes it, the risk of getting it, treatments.

We hear lots of information about things that are good for us, other things that are bad for us. I always pay attention to the ones that say chocolate and red wine is good for you. What do you do when you hear those kinds of things?

*Probes:*

Have you ever changed anything you do because of something you’ve heard in the news about health? Stopped doing something? Started something new? Doing something more or less often?

**Communication/Understanding about Liberation Therapy/venous angioplasty**

We would like to ask you some questions about Liberation Therapy/venous angioplasty—information you received or sought out, how good you thought that information was, and what you understood from the information you received.

What do you know about Liberation Therapy/venous angioplasty?

LIBERATION THERAPY/venous angioplasty: Liberation Therapy/venous angioplasty is an endovascular surgical procedure. LT involves inserting a tiny balloon with or without an artificial tube (i.e., a stent) into the internal jugular and/or azygous veins (that is, venous angioplasty), assumed to be blocked, to improve the impaired blood flow. For people doing this procedure, the belief is that it may reduce the symptoms of MS.

CCSVI (Chronic Cerebrospinal Venous Insufficiency) - is a term developed by Italian researcher Paolo Zamboni in 2008 to describe compromised flow of blood in the veins draining the central nervous system.

LIBERATION THERAPY and CCSVI: Zamboni hyposthesized that CCSVI plays a role in the cause of MS, and Liberation Therapy is the treatment to rectify the blockage.

If you want to learn something about your health, or a member of your family’s health, about something like Liberation Therapy or CCSVI, where do you go for information?

- Media (newspapers, including online sources, radio, television, programs like Dr. Oz, The Doctors)
- Internet (like government websites, the MS Society, general health sites (official or unofficial)
- Social networking sites (Facebook, MySpace, Twitter, blogs)
- HealthLinks / Telehealth
- Your doctor or other health professionals
- Friends or family
- Others?
  - How much did you trust that source of information? Why/why not?
  - How comfortable was your health care provider (your GP and/or neurologist) in discussing Liberation Therapy or CCSVI with you?
  - Were you confused by any of the messages that you heard?
    - Risks
    - Benefits

What kind of information would you like to know about Liberation Therapy or CCSVI?

How easy has it been to find information about Liberation Therapy or CCSVI?

Have you had the Liberation Therapy/venous angioplasty procedure?

Do you know someone who has had the Liberation Therapy/venous angioplasty procedure?

*For participants who have had Liberation Therapy/venous angioplasty:*

Do you think you were told everything about the benefits and risks of the procedure?

- From your health care provider?
- Did you look for information on your own?

How much did you trust the information that you found? Why/why not?

Were you confused by any of the messages that you heard?

- Risks
- Benefits

**Now that we have spoken a bit about some of these issues more generally, let’s get into something that has been more recently discussed**.

What have you heard in the news, if anything, about Canada’s policy position on Liberation Therapy or CCSVI in the last year?

NARRATIVE TO SHARE WITH PARTICIPANTS TO SET STAGE:

As many of you know, Liberation Therapy/venous angioplasty and chronic cerebrospinal venous insufficiency (CCSVI) have been covered quite controversially in the news. Scientists that do research and different kinds of doctors have been quoted as being in favour of the treatment, while others have been quoted as being against the treatment. Elected health officials have also kind of been all over the map on this issue. Initially, elected officials supported the position taken by the MS Society of Canada that the evidence that was initially coming out of Italy by a medical researcher named Zamboni should be taken with some caution. (In fact, the MS Society of Canada was quite vocally opposed to the Zamboni treatment because it was an untested therapy). Zamboni was hypothesizing that patients with MS have blocked veins that are impairing blood flow and that if you use Liberation Therapy/venous angioplasty, you can unblock those veins and improve a patient’s level of function. There have been a lot of before and after videos on the Internet of people with MS that have had the procedure; some showing remarkable improvements to their previous level of functioning. For other people, the video improvements haven’t been that remarkable. Some people have also died from complications associated with having Liberation Therapy/venous angioplasty.

So the initial position of the MS Society, and other doctors, many researchers and elected officials is that Liberation Therapy/venous angioplasty should be subjected to the same standard scientific principles that are followed in looking at any new kind of medical therapy/treatment. In this case, this would mean that we would need to wait until we have more studies that can assess if people with MS have blocked veins impairing blood flow that Zamboni was reporting, and also do many repeatable clinical trials to prove that Liberation Therapy/venous angioplasty both helps people with MS and is safe (the highest form of scientific evidence where some people are randomized to receive a treatment and some people are randomized into a control group and not receive the treatment). But, for some patients with MS and some medical researchers, this wait-and-see approach was not acceptable. They rallied together and applied a lot of pressure on elected officials. This prompted many different provincial governments to put money towards research, like these clinical trials, and/or to at least create a registry that will track the outcomes of patients with MS who go out of country to receive Liberation Therapy/venous angioplasty. The MS Society of Canada also changed its initial position and has put money towards researching this topic.

What do you think about the different positions that have been taken by:

- The government of Canada
- Your provincial government
  - In Manitoba, the provincial government has given $5M towards MS research on Liberation Therapy and CCSVI – specifically for a clinical trial. It is unclear if this is enough money to properly test Liberation Therapy.
- The MS Society of Canada
  - Initially the MS Society of Canada was vocally opposed to the idea of Liberation Therapy. Due to considerable pressure from MS patient groups, the MS Society of Canada is now presently reviewing and/or have funded research to look into Liberation Therapy/venous angioplasty and CCSVI to see if it is a safe and effective treatment for MS patients.

How should society make decisions about balancing how health research dollars are spent? For example, the government decision to add more money to research for MS and specifically Liberation Therapy and CCSVI takes away money away from other possible areas of research. This isn’t necessarily a bad thing, it is just the reality when you are dealing with limited pots of money.

So – how should society deal with these kinds of things?

What do you think are the most important issues here?

How should health policies be made when scientists have not reached a consensus about a particular care option? Are their times when it might be a better idea to set health policies before the evidence is known than other times?

Are you worried that this takes away available resources from other viable health pursuits?

What considerations should government policy makers (i.e. elected officials and health professionals that work for provincial health departments that advise elected officials) use to arrive at decisions about health when the evidence isn’t clear about what the best course of action to follow is?

What would you hope policymakers would be thinking about in making decisions about treatments like Liberation Therapy that hasn’t had enough good evidence about whether or not it works or is safe?

Would you hold the same position if it was for something else?

What criteria should policymakers use to make these decisions?

Concluding questions

If you had the ear of health policy-makers, what would you like to tell them about managing MS and Liberation Therapy/venous angioplasty?

Are there any final comments that you would like to make?
