# Supplementary material for: Caught in a no-win situation: discussions about CCSVI between persons with multiple sclerosis and their neurologists – a qualitative study
Source: BMC Neurol. 2017 Sep 7;17:176. doi: 10.1186/s12883-017-0954-7 (PMC5590111; doi:10.1186/s12883-017-0954-7)
Supplement: Supplementary file 3 — Key Informant (MS) Interview Questions. This document contains the semi-structured key informant interview guide used for clinicians, researchers, health policy makers, and advocacy organizations. Various individual tailoring for specific groups are represented in this document, but as interviews progressed, further tailoring was undertaken as most relevant for that key informant stakeholder group. (DOCX 26 kb) [file 12883_2017_954_MOESM3_ESM.docx]

**Key Informant Interview Questions**

**(modified for Multiple Sclerosis health care providers and decision-makers)**

**Introduction**

This research is looking at the process of policy development and decision making about treatments for diseases like Multiple Sclerosis when there is uncertainty, ambiguity, or controversy about some factor or factors crucial to making a sound, informed decision. We are particularly interested in the endovascular treatment (“Liberation Therapy”) for people with multiple sclerosis, which remains characterized by uncertainty:

We are also doing similar research concerning four current cancer control scenarios:

- mammography screening for women under the age of 50
- PSA screening
- HPV vaccine
- expensive cancer drugs

**General note on interview guide:** This guide covers the type of questions asked. Aside from the opening question, the order of the questions may change based on the nature of the conversation and what is raised first by the participant in terms of the topic. Things are explored to their natural conclusion or in terms of how it fits into another line of questioning contained within the guide. Hence the order listed below, while seemingly sequential, is intended as a topic area guide and not a prescriptive interviewing text.

**Questions**

1. To get us started, can you tell me about your background as [a neurologist/ vascular surgeon/ and advisor to decision makers] on Multiple Sclerosis /neurological disease treatment policy in Canada?

[if interviewing a decision-maker]

Can you tell me about your role and responsibilities [at your organization] as a decision maker on policies such as approval of treatments for diseases like Multiple Sclerosis in Canada?

1. Can you walk us through how decisions are made about what treatments are given to MS patients?

Can you give me an overview of how patient/ doctor / researcher input is incorporated into this process?

Probes:

Who are the key decision makers? Federally? Provincially?

Who are the major stakeholders, i.e., who plays a major role in influencing decision makers? Industry? Patient advocacy groups? Others?

Probes:

To what extent has the media played a role in how these decisions are being made? Are there any specific policies that have had to be implemented in recent years compared to prior protocol?

1. What do you see as the strengths of this process? What do you see as the challenges?

Probes:

Does that process change at all when there is uncertainty or ambiguity or doubt or controversy about some aspect of the policy?

How does it change what you might do?

Have there been many changes since 2009?

1. What would you characterize as the greatest challenges arising from uncertainty you (or your organization) face in making recommendations about new MS treatments?

Listen/probe for sources of uncertainty:

- evidence (e.g., effectiveness, safety, generalizability, transferability)
- costs, budget impact
- stakeholder interests/preferences (patients, clinicians)
- business influences (e.g., pharma lobbying for Gilenya, Betaseron or other drugs)
- political support
- clinical (individual) vs. population (societal) impacts

1. In your experience, what are the most common sources of uncertainty that you have had to deal with? What are the less common sources of uncertainty?

Listen/probe for sources of uncertainty:

- evidence (e.g., effectiveness, safety, generalizability, transferability)
- costs, budget impact
- stakeholder interests/preferences (patients, clinicians)
- business influences (e.g., pharma lobbying in betaseron, gilenya or other drugs)
- political support, clinical (individual) vs. population (societal) impacts

Also: to what extent if at all does uncertainty come from

- conflicting information coming from patients’ other health care providers?
- difficulty experienced in communicating with patients’ other health care providers?

1. What do you do to manage, navigate or mitigate uncertainty?

Probes:

What has worked well in helping you deal with your uncertainty?

Have you tried anything that didn’t work well?

Probe:

If participant talks only about formal tools (e.g., sensitivity analysis) or only about informal strategies (e.g., talking with peers), ask:

We’re interested in those ways of coping with uncertainty, but we are also interested in [more formal, sophisticated tools] or [more informal coping strategies] too. Do you use anything like that to help you deal with uncertainty?

Probe:

If participant does not mention tools that measure or quantify uncertainty, ask:

Do you ever use statistical techniques that measure or quantify the impact of uncertainty?

Probe for Health Care Providers (i.e. Neurologists or Vascular Surgeons):

What role does communication with your patients and/or your patients’ other health care providers play in how you mitigate uncertainty involved in treating your patients?

What methods of communication do you use and have you found most useful to give or get information to or from your patients and their families? (for example, brochures, peer reviewed journals, videos).

**NOTE: The following set of questions would come early or late in the interview depending on how the issue unfolded in the overall conversation. Hence, do not interpret that it’s placement at the end indicates that it wasn’t raised until the end.**

1. Let’s talk about the CCSVI issue. What did you see as the defining moments in the unfolding of this issue? What were your experiences?

Probe (for health care providers):

Was this something your patients came to you to discuss? How did you handle those conversations? What strategies did you use to manage these conversations? How well do you think you handled it given the uncertain nature of the issue?

What experiences from this were positive? Where there any negative experiences associated with this?

Probe (for health policy makers):

What challenges did you face in handling this issue given the extensive media coverage?

Did any concerns arise for you when other provincial jurisdictions (or federally) made decisions to fund (or not fund) clinical trials?

I did focus groups with some pwMS and they felt that even if the health system can’t fund the treatment, having the diagnostic tests (the scans to see if their veins are blocked) covered as an insured service should be made available. How do you react to that kind of situation? I would imagine that this isn’t the first time something like this has happened in a health system context.

1. That was my last question. Is there anything else that you would like to add about managing uncertainty in decision-making about new Multiple Sclerosis treatments?
2. Is there anyone else that you think it would be valuable for us to talk with about uncertainty and treatments for such variable diseases as Multiple Sclerosis?
