# Supplementary material for: Caught in a no-win situation: discussions about CCSVI between persons with multiple sclerosis and their neurologists – a qualitative study
Source: BMC Neurol. 2017 Sep 7;17:176. doi: 10.1186/s12883-017-0954-7 (PMC5590111; doi:10.1186/s12883-017-0954-7)
Supplement: Supplementary file 4 — Consolidated criteria for reporting qualitative research (COREQ) – Checklist. This document contains a checklist used when reporting qualitative research to ensure greater transparency around data collection, analysis, and reporting processes. (DOCX 22 kb) [file 12883_2017_954_MOESM4_ESM.docx]

| **No. Item** | **Guide questions/description** | **Reported on Page/Section** |
| --- | --- | --- |
| Domain 1: Research team and reflexivity |  |  |
| *Personal Characteristics* |  |  |
| 1. Interviewer/facilitator | Which author/s conducted the interview or focus group? | S. Michelle Driedger  Ryan Maier |
| 1. Credentials | What were the researcher’s credentials? | S. Michelle Driedger, PhD  Ryan Maier, MA |
| 1. Occupation | What was their occupation at the time of the study? | SMD:Associate professor  RM: Qualitative research analyst |
| 1. Gender | Was the researcher male or female | SMD: female  RM: male |
| 1. Experience and training | What experience or training did the researcher have? | SMD is an instructor of qualitative methods and has extensive experience in focus group/interview data collection and analysis.  RM is an experienced qualitative analyst and had previous experience and training (by SMD) in assisting and facilitating focus groups and interviews. |
| *Relationship with participants* |  |  |
| 1. Relationship established | Was a relationship established prior to study commencement? | No |
| 1. Participant knowledge of the interviewer | What did the participants know about the researcher? E.g. personal goals, reasons for doing the research | Participants were provided with an information sheet about the research and provided informed consent to participate in the study. Participants were recruited through a research coordinator affiliated with the local MS Clinic through a registry of patients having already consented to being contacted to participate in research studies. This is described in more detail in the additional Methods file. |
| 1. Interviewer characteristics | What characteristics were reported about the interviewer/facilitator? E.g. bias, assumptions, reasons and interest in the research topic | Participants were provided with an information sheet (for informed consent) which included statements about what the research project was about (investigating health decision making in situations of uncertainty) and listing of funding organization (Multiple Sclerosis Society of Canada). |
| **Domain 2: study design** |  |  |
| *Theoretical framework* |  |  |
| 1. Methodological orientation and theory | What methodological orientation was stated to underpin the study? e.g. grounded theory, discourse analysis, ethnography, phenomenology, content analysis | Methods; Additional File 1 – Detailed Methods |
| *Participant selection* |  |  |
| 1. Sampling | How were participants selected? e.g. purposive, convenience, consecutive, snowball | Methods; Additional File 1 – Detailed Methods |
| 1. Method of approach | How were participants approached? e.g. face-to-face, telephone, mail, email | Methods; Additional File 1 – Detailed Methods |
| 1. Sample size | How many participants were in the study | Methods; Additional File 1 – Detailed Methods |
| 1. Non-participation | How many people refused to participate or dropped out? Reasons? | No participants refused to participate in the study or dropped out. |
| *Setting* |  |  |
| 1. Setting of data collection | Where was the data collected? E.g. home, clinic, workplace | Focus Groups: Data collected at an accessible room at the University of Manitoba, Winnipeg, Canada  Key Informants: Data collected at sites of mutual convenience, either in person or by telephone. Most interviews were conducted by the lead researcher (SMD) in Winnipeg, Manitoba, Canada, with some in person interviews in Vancouver, British Columbia, Canada (while at a national MS educational training opportunity where some Key Informants were also attending). |
| 1. Presence of non-participants | Was anyone else present besides the participants and researchers? | No |
| 1. Description of sample | What are the important characteristics of the sample? e.g. demographic data, date | Methods; Additional File 1 – Detailed Methods |
| *Data collection* |  |  |
| 1. Interview guide | Were questions, prompts, guides provided by the authors? Was it pilot tested? | Methods; Additional File 1 – Detailed Methods |
| 1. Repeat interviews | Were repeat interviews carried out? If yes, how many? | No |
| 1. Audio/visual recording | Did the research use audio or visual recording to collect the data? | Methods; Additional File 1 – Detailed Methods |
| 1. Field notes | Were field notes made during and/or after the interview/focus group? | Yes |
| 1. Duration | What was the duration of the interviews/focus groups | Methods |
| 1. Data saturation | Was data saturation discussed? | Additional File 1 – Detailed Methods |
| 1. Transcripts returned | Were transcripts returned to participants for comment and/or for correction? | No. However, during the informed consent process, participants were told that they could contact the researchers at any time and request any changes to their comments that they wished. They were also provided with a brief report about the key themes that arose during the focus groups (with quotes included) and they were informed that they could revise or contest anything that they found in the report that they disagreed with. The only participant feedback received from this ‘returning of results’ report was positive and that they felt we had captured their sentiments. |
| **Domain 3: analysis and findings** |  |  |
| *Data analysis* |  |  |
| 1. Number of data coders | How many data coders coded the data? | Methods; Additional File 1 – Detailed Methods |
| 1. Description of the coding tree | Did the authors provide a description of the coding tree? | Methods; Additional File 1 –Methods; Additional File 1 – Detailed Methods |
| 1. Derivation of themes | Were themes identified in advance or derived from the data? | Methods; Additional File 1 – Detailed Methods |
| 1. Software | What software, if applicable, was used to manage the data? | Methods; Additional File 1 – Detailed Methods |
| 1. Participant checking | Did participants provide feedback on the findings? | Participants were given a report on the key themes that arose during all focus groups (including those found in this article) and with that report they were given the contact information of the researchers in case they wished to provide further feedback on findings. No participants attempted to provide further feedback beyond some positive comments expressed after receipt of the report. |
| *Reporting* |  |  |
| 1. Quotations presented | Were participant quotations presented to illustrate the themes/findings? Was each quotation identified? E.g. participant number | Results |
| 1. Data and findings consistent | Was there consistency between the data presented and the findings | Discussion |
| 1. Clarity of major themes | Were the major themes clearly presented in the findings? | Results |
| 1. Clarity of minor themes | Is there a description of diverse cases or discussion of minor themes | Results |

Checklist derived from:

Tong A, Sainsbury P, Craig J. Consolidated criteria for reporting qualitative research (COREQ): a 32-item checklist for interviews and focus groups. Int J Qual Health Care. 2007;19(6):349-57. doi: 10.1093/intqhc/mzm042
